# Cycloserine inhibits glutamate decarboxylase from *Mycobacterium tuberculosis*

**DOI:** 10.1080/14756366.2026.2707854

**Published:** 2026-09-21

**Authors:** Jan Snášel, Jiří Dostál, Michal Tupec, Ondřej Bulvas, Iva Pichová

**Affiliations:** Institute of Organic Chemistry and Biochemistry, Czech Academy of Sciences, Prague, Czech Republic

**Keywords:** Glutamate decarboxylase, *Mycobacterium tuberculosis*, cycloserine, PLP-oxime

## Abstract

Glutamate decarboxylase (GadB) is a pyridoxal phosphate (PLP)-dependent enzyme that contributes to intracellular pH homeostasis and supports the survival of *Mycobacterium tuberculosis* (*Mtb*) in lung tissue, granulomas, and within host macrophages. Here, we demonstrate that glutamate decarboxylase from *Mycobacterium tuberculosis* (*Mtb*GadB) is a multimeric enzyme exhibiting maximal activity under acidic conditions. *Mtb*GadB is inhibited by the clinical antibiotic d-cycloserine as well as l-cycloserine *in vitro*. Both cycloserine enantiomers break the internal aldimine bond between PLP and the conserved Lys277 residue, forming a previously unrecognised PLP-oxime product and subsequently inhibiting catalysis. Given that PLP-dependent enzymes are promising drug targets, understanding the chemical behaviour of PLP is essential for developing selective inhibitors.

## Graphical abstract

**Figure:**
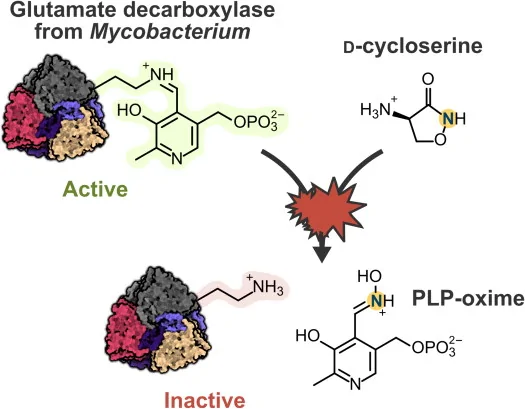

## Introduction

*Mycobacterium tuberculosis* (*Mtb*) is the etiologic agent of tuberculosis (TB), which remains one of the most prevalent and deadly infectious diseases worldwide^1–3^. To survive within host macrophages and maintain essential metabolic processes, *Mtb* must tightly regulate its intracellular pH while residing in phagosomal compartments destined for acidification as part of the host defense response. Although mature phagolysosomes are typically acidic, *Mtb* can interfere with phagosome maturation and acidification, enabling the bacterium to survive within a relatively less acidic intraphagosomal environment^4–6^. Adaptation to acid stress, crucial for *Mtb* intracellular persistence, is associated with extensive metabolic remodelling and pH-dependent growth phenotypes^4^^,^^7^.

Glutamate decarboxylase (GAD; EC 4.1.1.15) is a pyridoxal phosphate (PLP)-dependent lyase enzyme that catalyses the irreversible decarboxylation of l-glutamate (l-Glu) to γ-aminobutyrate (GABA), consuming a proton and releasing CO_2_^8^^,^^9^_._ In bacteria, GAD is a key component of the glutamate-dependent acid resistance (GDAR) system, facilitating survival under extremely acidic conditions. During glutamate decarboxylation, the GDAR system consumes intracellular protons and exports GABA in exchange for glutamate via a dedicated glutamate/GABA antiporter (GadC), which helps buffer the cytoplasm and maintain intracellular pH^10^. The GDAR system has been well documented in organisms such as *Escherichia coli*, which encodes the GadA/GadB decarboxylases and the GadC antiporter within the GDAR regulon^11^, and in *Listeria monocytogenes*, where acid resistance is mediated by GadD/GadT proteins, mainly by the *gadT2*/*gadD2* cluster^12^^,^^13^.

One of the proposed acid resistance mechanisms involved in *Mtb* is *Mtb*GadB, a GAD enzyme encoded by the *gadB* gene (Rv3432c)^14^^,^^15^. Until recently, this enzyme was not considered a functional contributor to mycobacterial acid resistance, in part because the canonical enterobacterial *gadA*/*gadB*/*gadC*-type GDAR system is not clearly conserved in *Mtb*^11^^,^^14^. However, accumulating evidence indicates that GadB is expressed and active under acidic conditions (low pH values), promoting survival within macrophages^16^. In mycobacteria, genetic disruption of GAD function reduces survival under acidic conditions, whereas complementation restores acid tolerance, pointing to the protective role of GABA production under acid stress^16^. Accordingly, the heterologous expression of *Mtb*GadB in *Mycobacterium bovis* improves bacterial survival within macrophages, suggesting that GadB-dependent GABA production contributes to resistance against intraphagosomal stress^16^.

Beyond its role in acid resistance, GadB may contribute to mycobacterial metabolism through the GABA shunt. This pathway converts glutamate to succinate via GABA and succinate-semialdehyde, potentially supporting anaplerosis. This hypothesis is supported by research on the presence of succinate-semialdehyde dehydrogenase GabD1 within *Mtb*^17^ and by the known metabolic plasticity of the bacterium (reviewed in Ref.^18^).d-cycloserine (DCS) is an antibiotic historically used as a second-line treatment for tuberculosis. Structurally, DCS is a cyclic mimetic of d-alanine. Its primary antimicrobial activity results from inhibition of two essential enzymes involved in peptidoglycan biosynthesis: alanine racemase (Alr) and d-alanine:d-alanine ligase (Ddl)^19^. Metabolomics-based studies in mycobacteria indicate that Ddl is the principal lethal target under cellular conditions, whereas Alr inhibition contributes indirectly by limiting d-alanine availability and thereby modulating Ddl inhibition^19–21^. At the molecular level, DCS inhibits Alr by interrupting its internal aldimine bond with PLP, forming either a tightly binding 3-hydroxy-4-isoxazolyl-substituted pyridoxamine phosphate (PMP-isoxazole) or a weakly binding 2-aminopropanoate-substituted PLP-oxime^22^^,^^23^. Beyond these canonical targets, DCS and its enantiomer l-cycloserine (LCS) may act as mechanism-based inhibitors of other PLP-dependent enzymes in *Mtb*, such as the branched-chain aminotransferase IlvE, for which a cycloserine-inhibited crystal structure has been reported^24^.

Here, we demonstrate that *Mtb*GadB remains fully active *in vitro* under acidic conditions and is inhibited by the clinical antibiotic DCS and its enantiomer LCS. Using fluorescence spectroscopy, GC-MS analysis of the inhibition reaction, and X-ray crystallography, we show that this inhibition interrupts the internal aldimine bond between the PLP coenzyme and the conserved Lys277 residue, forming an unreactive adduct PLP-oxime. These findings establish *Mtb*GadB as an additional PLP-dependent enzyme susceptible to cycloserine *in vitro*.

## Materials and methods

### Sequence analysis

Multiple sequence alignment was generated from GAD sequences retrieved from public databases (Supplemental Table S1) using Muscle5^25^. Sequence similarity was calculated using the Sequence Manipulation Suite^26^.

### Cloning, expression, and purification of *Mtb*GadB

The *Mtb*GadB protein-coding sequence (Rv3432c)^27^ was cloned from genomic DNA of the *Mtb* strain H37Rv (ATCC) into the pET-28b vector, providing an *N*-terminal thrombin-cleavable His-tag. The K277R mutation was introduced into the *gadB* sequence using primers GadB_F and 277 R_R, and 277 R_F and GadB_R (Supplemental Table S2) to generate two overlapping fragments. These fragments were subsequently fused by a second PCR. Restriction sites were introduced using primers NdeI_F and XhoI_R, followed by digestion with *Nde*I and *Xho*I enzymes (New England Biolabs) and ligation into the vector. Construct integrity was verified using Sanger sequencing (Eurofins Genomics).

After transformation into *E. coli* BL21(DE3), cells were grown in nutrient broth (HiMedia, Supelco) containing kanamycin (100 µg/mL) at 30 °C to an OD_600_ of approximately 0.4. Protein expression was induced with 1 mM IPTG, and cultures were incubated overnight at 25 °C. Cells were harvested in lysis buffer (50 mM Tris–HCl, pH 8.0, 500 mM NaCl) supplemented with protease inhibitor cocktail (Roche), treated with lysozyme (0.5 µg/mL), and sonicated on ice. After centrifugation (20,000 × g, 30 min, 4 °C), the supernatant was applied to an Ni–NTA agarose (Qiagen) column. The column was washed with lysis buffer, followed by lysis buffer containing 20 mM imidazole. His-tagged *Mtb*GadB was eluted with 500 mM imidazole. The protein was dialysed against 50 mM Tris–HCl (pH 8.0) containing 5 mM β-mercaptoethanol and further purified by size-exclusion chromatography using a Superdex 200 10/300 GL column (Cytiva). Purified protein aliquots were stored at −80 °C.

For native *Mtb*GadB, His-tag cleavage was performed using biotinylated thrombin (10 U; Sigma-Aldrich) in the presence of 1 mM CaCl_2_ for 4 h at room temperature. Thrombin was removed using a Streptavidin HP SpinTrap column (GE Healthcare). The *Mtb*GadB protein was concentrated to 20 mg/mL using Amicon Ultra 30 kDa filters (Millipore) and stored at −80 °C.

### Mass photometry

Mass photometry was performed using a TwoMP instrument (Refeyn) calibrated with bovine serum albumin and immunoglobulin G standards (15 nM) in 50 mM citrate–phosphate buffer (pH 7.1). *Mtb*GadB (20 nM) was analysed in 50 mM citrate–phosphate buffer (pH 4.4–7.8). Movies were recorded for 60 s and analysed using DiscoverMP software (Refeyn).

### PLP removal

The PLP coenzyme was released from the protein by incubating *Mtb*GadB in 100 mM Tris–HCl (pH 8.6) or 100 mM potassium phosphate (pH 7.2) containing 5 mM hydroxylamine (HA) for 24 h at 4 °C. Excess PLP was removed by dialysis against 50 mM Tris/HCl (pH 8.6) with 2.5 mM dithiothreitol.

### GAD activity assay

Primary GAD reaction mixtures contained 0.2 M sodium acetate (pH 4.5) and 10 µM *Mtb*GadB in a total volume of 200 µL. Reactions were initiated by adding l-glutamate (l-Glu; 0–17.5 mM final concentration), incubated for 30 min at room temperature, and stopped by boiling for 5 min. GABA concentration was subsequently determined using one of the following two procedures.

(1) *GABase-coupled spectrophotometry*: The secondary GABAase reaction mixture contained 0.5 M potassium pyrophosphate (pH 8.8) with 4 mM NADP^+^, 20 mM α-oxoglutarate, and GABAase (10 U/mL; mixture of GABA transaminase and succinic semialdehyde dehydrogenase from *Pseudomonas fluorescens*, all obtained from Sigma-Aldrich). The boiled primary reaction (100 µL) was added to 200 µL of the secondary reaction mixture and incubated overnight at room temperature. Absorbance at 340 nm was measured using a Tecan Infinite M1000 plate reader (Tecan). Activities were normalised to maximal activity (relative activity scale) and plotted using SigmaPlot v11.0 (SYSTAT Software Inc.). All assays were performed in triplicate.

(2) *Colorimetric visualisation*: Alternatively, the amine (GABA) produced by *Mtb*GadB was quantified using a modified Berthelot reaction^28^ performed by thoroughly mixing 0.5 mL of the boiled sample with 0.2 mL of 0.2 M borate buffer and 1 mL of 6% (w/v) phenol. After cooling on ice, 0.4 mL of 5–10% (w/v) NaOCl was added, followed by cooling on ice. Samples were boiled for 10 min and then cooled; absorbance was measured at 630 nm using ethanol as a blank sample.

### Effects of pH, ions, and potential inhibitors on activity

*Mtb*GadB (10 µM) was incubated as described below, followed by boiling and GABase assay. All reactions were carried out for 30 min.

For pH dependence, *Mtb*GadB activity was measured in 50 mM citrate–Bis-tris propane buffer at pH values ranging from 4.5 to 8.0 (0.5 pH increments) with 1 mM l-Glu.

For screening of metal or ammonium ion effects, *Mtb*GadB in 200 μL was preincubated for 30 min with metal (Mg^2+^, Mn^2+^, Zn^2+^, Ca^2+^, Cu^2+^; 0–20 mM) or ammonium (10 μL 0–50 mM) cations in 0.2 M sodium acetate (pH 4.5). Reactions were initiated with 1 mM l-Glu. Activity in the absence of additional ions was defined as 100%.

### Dependence of activity on enzyme concentration

*Mtb*GadB was added to 200 μL aliquots of 200 mM sodium acetate (pH 4.5) containing 1 mM l-Glu to achieve concentrations of 20, 30, 60, and 150 μg/mL. Reactions proceeded for 30 min at room temperature, followed by boiling and GABase assay.

### Substrate specificity screening

Substrate specificity was tested using the 20 standard l-amino acids, d-glutamate (d-Glu), and d-glutamine (d-Gln) (20 mM each). *Mtb*GadB (10 µM) was incubated in 0.2 M sodium acetate (pH 4.5) in the presence of 0.5 mM l-Glu for 30 min. Reactions were terminated by boiling and analysed by colorimetric visualisation.

### Inhibition by cycloserine

*Mtb*GadB (10 µM) in 0.2 M sodium acetate buffer (pH 4.5, 200 μL total volume) was incubated with 10 μL d-cycloserine (DCS; 2–10 mM) or l-cycloserine (LCS; 0.6–2.0 mM). Reactions were initiated with l-Glu (0.1–17.5 mM), incubated for 30 min, stopped by boiling, and analysed by GABase assay.

### Fluorescence spectroscopy

To detect the internal aldimine, 100 µM *Mtb*GadB was analysed in 20–50 mM sodium phosphate buffer (pH 5.5–6.0). Fluorescence spectra (300–550 nm) were recorded upon excitation at 434 nm using a Tecan Infinite M1000 plate reader (Tecan)^8^^,^^9^.

### Hydroxylamine assay

Hydroxylamine (HA) in DCS samples was quantified using an indirect spectrophotometric assay as previously described^29^. Aliquots of samples or HA standards (1 μM–1 mM, positive control) were transferred to 10 mL volumetric flasks containing 6 mL of distilled water. Subsequently, 1 mL of 0.047 M iodate solution was added, followed by 1 mL of 3 M sulphuric acid. The mixture was allowed to stand for 5 min at room temperature, after which 2 mL of 0.0035 M neutral red solution was added. The reaction was monitored spectrophotometrically by measuring the change in absorbance at 525 nm after several minutes.

### Product silylation and GC*-*MS analysis

The product of *Mtb*GadB inhibition was determined by GC-MS following trimethylsilylation. Briefly, one aliquot of 10 µM *Mtb*GadB was mixed with two aliquots of 200 mM sodium acetate (pH 4.5) to achieve a final pH range of 5.5–6.0. After removing precipitate by centrifugation, either l-cycloserine, d-cycloserine, *rac*-[2-^15^N-4,5,5-^2^H_3_]-cycloserine (^15^N-CS, LGC Standards), or HA was added to achieve a final concentration of 2 mM (1 mM for HA). The mixture was incubated on ice for 1 h and then lyophilised. The residue was derivatized with 40 μL BSTFA/TMCS reagent (99:1, TCI Europe) and 80 μL acetonitrile for 30 min at 70 °C. The supernatant (3 μL) was injected (splitless inlet, 250 °C) onto a TG-5MS column (30 m × 250 μm ID × 0.25 μm film thickness; Thermo Fisher Scientific), installed in a TRACE 1310 gas chromatograph coupled to an ISQ LT mass spectrometer (Thermo Fisher Scientific). After 1 min at 80 °C, the oven temperature was increased to 340 °C at 8 °C/min, followed by a 3 min hold.

The *m*/*z* values of silylated PLP-oxime molecular ions were calculated as weighted averages over 549 < *m*/*z* > 556, using signal intensity as the weighting factor and reported as mean values.

### Protein crystallisation and data collection

*Mtb*GadB was concentrated to 20 mg/mL in 50 mM Tris–HCl buffer (pH 8.0) containing 5 mM β-mercaptoethanol. Initial screening (PEGs Suite I, JCSG Core Suite I, Morpheus screen; Molecular Dimensions) used sitting-drop vapour diffusion (1:1 protein:reservoir solution ratio) in 96-well plates (Hampton Research) using a mosquito LCP robot (Molecular Dimensions). Initial protein concentrations were 15 mg/mL and 7.5 mg/mL. Optimised crystals were obtained at 7.5 mg/mL protein in 0.1 M sodium acetate buffer (pH 5.0) containing 20% PEG 200.

For soaking experiments, *Mtb*GadB crystals (200 µm) were preincubated in 0.1 M sodium acetate (pH 5.0) containing 20% PEG 200 for 1 h, followed by the addition of either 1 mM DCS or LCS, 1 h soaking, cryoprotection in mother liquor, and flash-freezing.

Diffraction data were collected at 100 K on a beamline at the PETRA III synchrotron radiation storage ring facility (DESY, Hamburg; wavelength 0.97625 Å) or on beamline 14.1 at the BESSY II third-generation synchrotron radiation source (Helmholtz-Zentrum Berlin; wavelength 0.9184 Å), both achieving a resolution of 1.4 Å.

### Structure determination

Data integration and scaling were performed using XDS^30^. Structures were solved by molecular replacement using *E. coli* GadB (PDB 1PMM) as the search model. The initial model was obtained with Phaser v2.8.3^31^ and further refined using phenix.refine (Phenix package v1.20.1–4487)^32^ and manual model building (Coot v0.9.8.7)^33^. Statistics for data collection, processing, structure solution, and refinement were calculated with phenix.table_one. Structural figures were generated with PyMOL Molecular Graphics System v2.5.4 (Schrödinger) and UCSF ChimeraX 1.10^34^.

## Results

### *Mtb*GadB remains active under acidic pH conditions

*Mtb*GadB (NCBI accession WP_003418277) is a 460-amino acid protein sharing 60.5%, 59.8%, and 54.9% sequence similarity with GADs from *E. coli*, *Levilactobacillus brevis*, and *Bacteroides thetaiotaomicron*, respectively (Supplemental Figure S1). The PLP coenzyme putatively binds to the conserved Lys277 residue.

**Figure 1.**
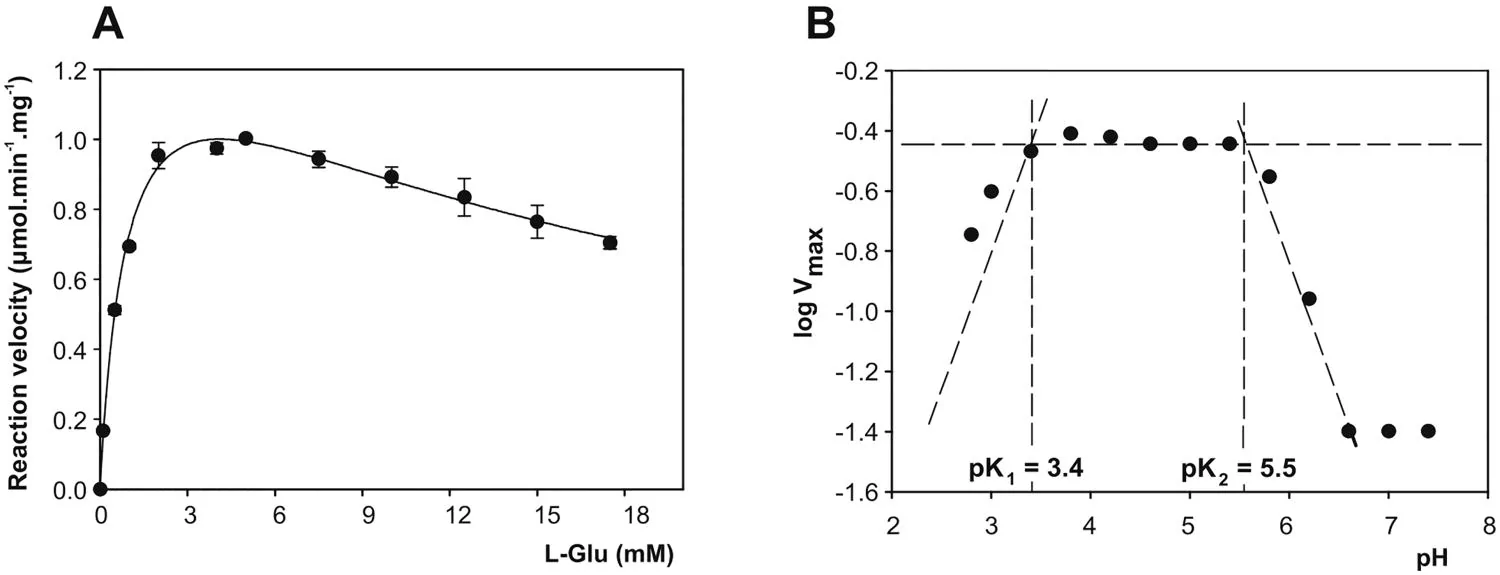
Biochemical characterisation of *Mtb*GadB. (A) Dependence of enzymatic activity on l-glutamate concentration. Data were fitted with the Briggs–Haldane kinetic model. (B) pH-dependent activity profile determined in citrate–Bis-tris propane buffer, indicating approximate pK values.

To establish the role of *Mtb*GadB in the response to acidic conditions, we first performed a biochemical characterisation of the enzyme. The enzyme was heterologously expressed in *E. coli* and purified by chromatography (Supplemental Figure S2A), followed by removal of the *N*-terminal His-tag (approximate yield 20 mg native GadB per litre culture). *Mtb*GadB decarboxylase activity was then assayed using l-Glu as the substrate, confirming the enzyme as catalytically active (Supplemental Figure S2B). The Briggs**–**Haldane kinetic model best describes the dependence of the initial reaction velocity on l-Glu concentration (Figure 1(A), *K*_M_ = 1.0 ± 0.1 mM), where the enzyme exhibits moderate substrate inhibition (*K*_i_ = 17 ± 2 mM). Beyond l-Glu, *Mtb*GadB does not convert any of the other standard amino acids, d-glutamate, or d-glutamine (data not shown), demonstrating strict specificity for l-Glu and the absence of glutaminase activity. The enzyme is largely insensitive to NH_4_^+^, Mg^2+^, Mn^2+^, Ca^2+^, and Zn^2+^ ions, whereas Cu^2+^ inhibits activity at millimolar concentrations (Supplemental Figure S3, data not shown).

The pH dependence of *Mtb*GadB activity was assessed within a pH range of 3 to 8. Maximal activity was observed between pH values of 3.5 and 5.5, resulting in a relatively broad plateau across the activity profile (Figure 1(B)). Enzyme activity declines markedly at pH values above 6.0 and below 3.5. This pH optimum is comparable to those reported for GADs from other species (Supplemental Table S3). In addition, the oligomeric state of *Mtb*GadB under different pH conditions was examined using mass photometry. At acidic pH values (4.5–6.0), *Mtb*GadB predominantly exists as a mixture of dimers and hexamers, whereas increasing pH towards 6.0 shifts the equilibrium in favour of a higher proportion of dimers (Supplemental Figure S4). However, the relationship between the activity and oligomeric state of *Mtb*GadB remains unclear.

### Cycloserine enantiomers inhibit *Mtb*GadB

DCS is known to inhibit two essential PLP-dependent enzymes involved in (myco)bacterial cell wall biosynthesis: alanine racemase and d-alanine:d-alanine ligase^19^^,^^23^. We therefore hypothesised that *Mtb*GadB, which also utilises PLP as a coenzyme, might be inhibited by DCS, LCS, or both enantiomers.

**Figure 2.**
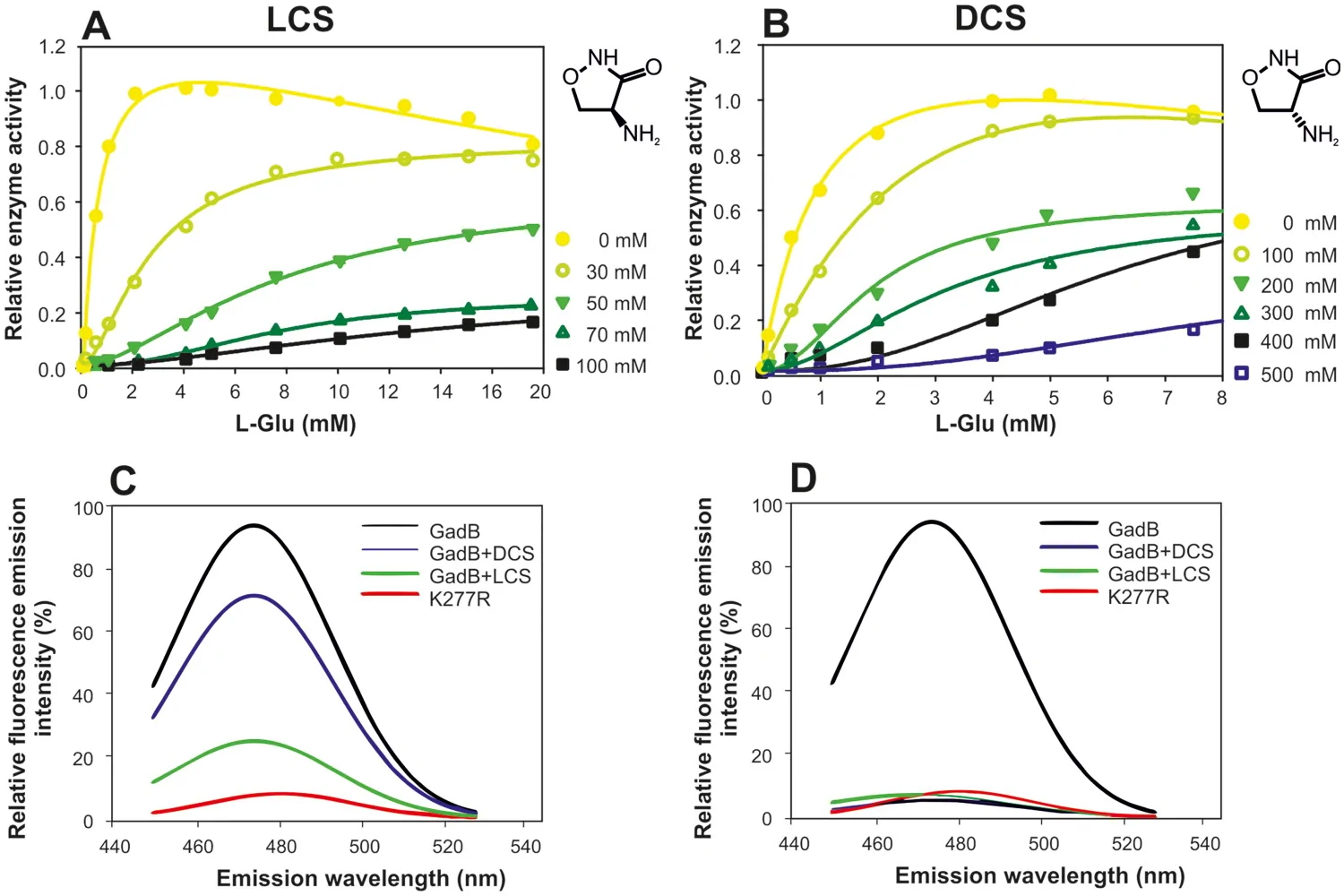
Inhibition of *Mtb*GadB by cycloserine enantiomers. (AB) Effect of (A) l-cycloserine (LCS) and (B) d-cycloserine (DCS) on *Mtb*GadB activity. Enzyme activity was measured at increasing l-glutamate (l-Glu) concentrations in the presence of the indicated concentrations of LCS or DCS. The highest activity observed for each inhibitor experiment was normalised to 100%. (CD) Fluorescence emission spectra of *Mtb*GadB after the addition of LCS or DCS recorded immediately (C) and after 1 h incubation at room temperature (D). A catalytically inactive K277R mutant served as a positive control for the absence of the PLP-aldimine.

Enzyme inhibition assays confirmed that both enantiomers inhibit the enzyme (Figure 2(A,B)**)**. Notably, increasing concentrations of either inhibitor alters the dependence of *Mtb*GadB activity on l-Glu concentration from a hyperbolic to a sigmoidal profile. Under saturating substrate conditions, LCS inhibits *Mtb*GadB with an IC_50_ of 36 μM, whereas DCS is less potent, having a ninefold-higher IC_50_ value.

The inhibition process was further monitored using fluorescence spectrometry, as loss of the internal aldimine in PLP-dependent enzymes is known to result in a marked decrease in fluorescence emission^8^^,^^9^. Confirming this assumption, incubation of *Mtb*GadB with cycloserine enantiomers led to a time-dependent decrease in fluorescence intensity (Figure 2(C,D)). As a positive control, we used a Lys277Arg mutant devoid of the internal aldimine. The more rapid reduction in fluorescence observed in the presence of LCS (Figure 2(C)) correlates with the greater potency of LCS-mediated inhibition compared to DCS (Figure 2(A,B)).

### Structure of native *Mtb*GadB confirms hexameric assembly and the internal aldimine

To elucidate the structural basis of *Mtb*GadB inhibition by cycloserine, we determined high-resolution crystal structures of both the native enzyme and its DCS-treated variant. *Mtb*GadB was crystallised at pH 5.0 under conditions that preserve its catalytic activity. The enzyme formed crystals in the R32 space group; both structures were refined to a resolution of 1.4 Å (Supplemental Table S4).

**Figure 3.**
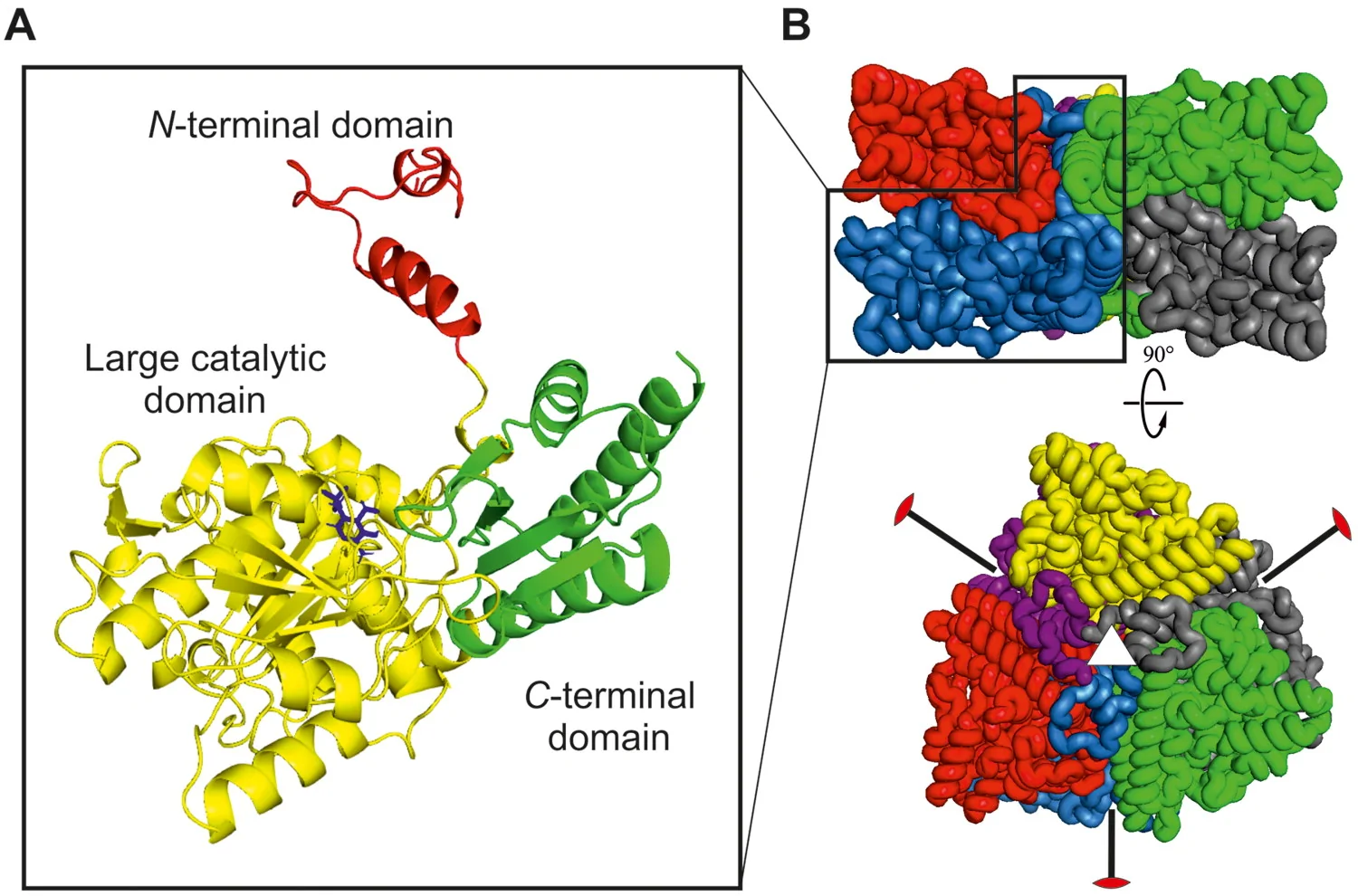
Structure of *Mtb*GadB. (A) Cartoon representation of the *Mtb*GadB monomer, highlighting its three domains: the N-terminal domain (red), the large catalytic domain (yellow), and the C-terminal domain (green). The catalytic domain contains the bound pyridoxal phosphate (PLP), shown in stick representation (dark blue). (B) Oligomeric structure of *Mtb*GadB illustrating its hexameric assembly; each subunit is shown in a different colour. The threefold symmetry axis is indicated by a white triangle; the twofold symmetry axes are represented by black lines terminating in red ellipses.

Each *Mtb*GadB monomer consists of three major domains: an *N*-terminal regulatory domain (residues ∼1–57), a large catalytic PLP-binding domain (residues ∼58–346), and a *C*-terminal domain (residues ∼347–460) (Figure 3(A)), consistent with published structures^35^. The active site is located at the interface between the catalytic and N-terminal domains of the same monomer. It also contains residues from the neighbouring subunit within the dimer, including the conserved β-hairpin (Supplemental Figure S5A), a common feature of group II decarboxylases^35–38^. Residues 1–13 and 447–460 were not visible in the electron density, indicating flexibility or disorder at both termini.

The enzyme assembles into a hexamer composed of six identical subunits (306 kDa in total), arranged as a trimer of dimers in a two-tiered, doughnut-like architecture featuring 32 point-group symmetry (Figure 3(B)). This oligomeric organisation is characteristic of bacterial GADs, consistent with structures determined under acidic conditions^35^^,^^38^.

**Figure 4.**
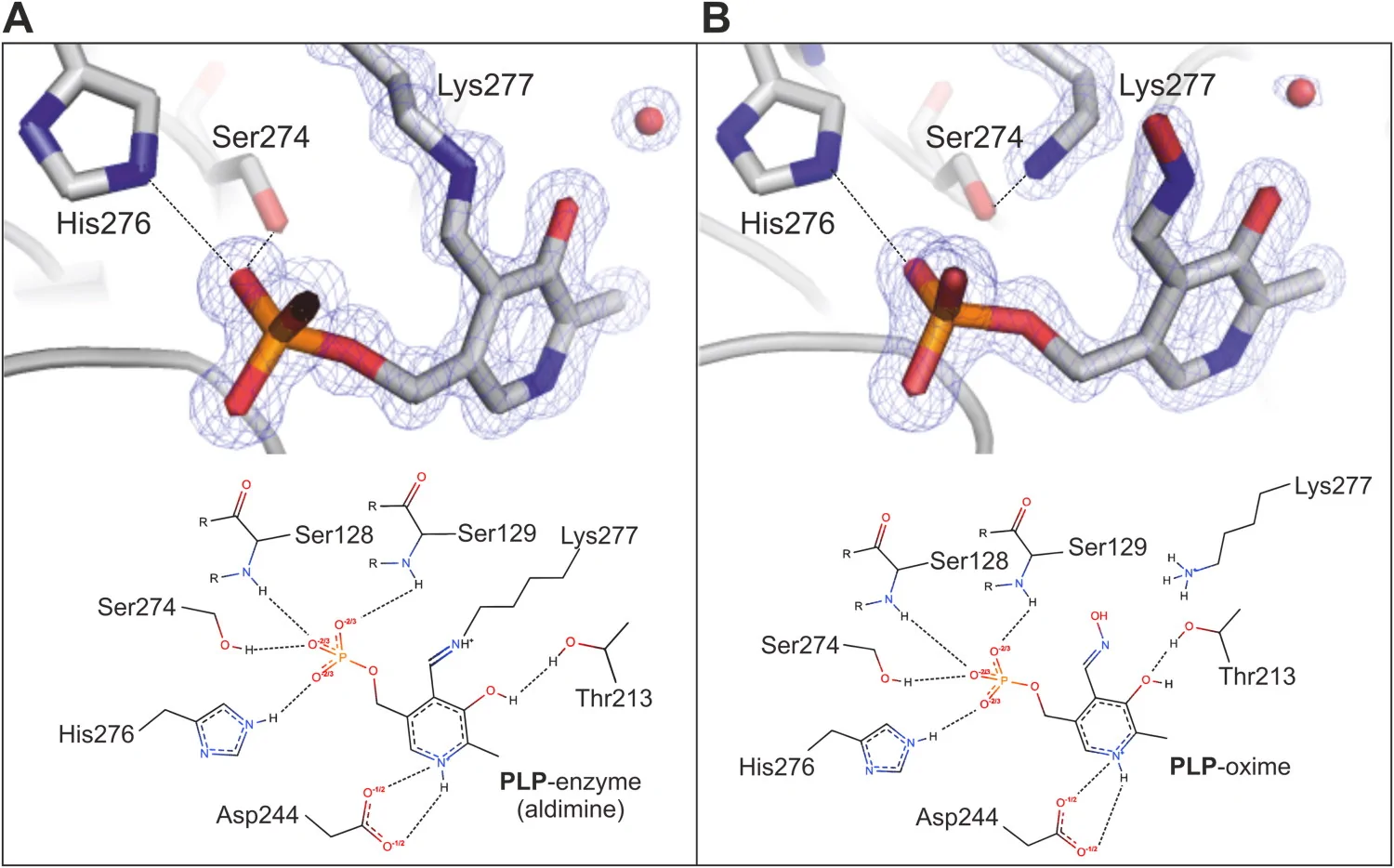
Effect of DCS treatment on the *Mtb*GadB active site. (A) Active site of native *Mtb*GadB with PLP covalently linked to the Lys277 residue, forming an internal aldimine. (B) Active site of *Mtb*GadB after DCS treatment, indicating internal aldimine cleavage and the presence of a PLP derivative, presumably PLP-oxime. The 2Fo-Fc electron density maps are contoured at 1.0σ. The lower part shows PoseView 2D interaction diagrams for the PLP–Lys277 internal aldimine and PLP-oxime, respectively. Dashed lines indicate hydrogen bonding.

In the native structure, the PLP coenzyme is clearly resolved in the active site, forming an internal aldimine with the ε-amino group of the Lys277 residue (Figure 4(A)). At 1.4 Å resolution, the electron density unambiguously defines the PLP–Lys277 linkage as well as the interactions stabilising the coenzyme. The phosphate group of PLP is anchored by hydrogen bonds with Ser128, Ser129, and His276, while the pyridine ring engages in stacking and hydrogen-bonding interactions with various residues, including Asp244 and Thr213 (Figures 4(A) and Supplemental S5B). Additional conserved active site residues (such as Phe62) line the PLP-binding pocket in a manner similar to other group II decarboxylases^36^, underscoring the conserved nature of the active site architecture.

### Structure of DCS-treated enzyme reveals a PLP-like derivative

In contrast to the native enzyme, the structure of *Mtb*GadB incubated with DCS reveals absence of the internal PLP–Lys277 aldimine. In this structure, no connecting electron density is observed between the Lys277 ε-amino group and the C4’ carbon of PLP. This indicates the elimination of Lys277 from the former aldimine bond with PLP and its subsequent rearrangement (Figure 4(B)). Despite this local rearrangement, the overall protein structure remains essentially unchanged, with an RMSD of approximately 0.32 Å over 433 C_α_ atoms compared to the native enzyme. The hexameric assembly is fully preserved, suggesting that the effect of DCS is localised to the active site.

While PLP-like electron density remains in the active site after cleavage of the aldimine bond, its altered shape suggests that PLP undergoes a chemical modification at the C4’ carbon (Figure 4(B)). Unlike native PLP, in which the C4’ carbon is bonded to only one nonhydrogen atom (the aldehyde oxygen), the modified PLP in the DCS-treated enzyme can be modelled with an additional nonhydrogen substituent attached at the C4’ position. This limitation of atom number rules out the presence of the previously reported DCS-derived PMP-isoxazole and 2-aminopropanoate-substituted PLP-oxime^23^. We therefore hypothesised that the unknown moiety could be an aldoxime (C4’=NOH) originating from DCS. Under this arrangement, the putative oxime occupies a position similar to that of native aldimine, positioned to form plausible hydrogen bonds with nearby residues or ordered water molecules (Figure 4(B)). Although bound in the active site, the PLP-oxime likely cannot form a covalent bond with the Lys277 residue, thereby blocking catalysis.

### Reactions of *Mtb*GadB with cycloserine generate PLP-oxime

To identify the PLP derivative observed in the *Mtb*GadB active site in DCS-soaked crystals, we incubated the enzyme with LCS, DCS, or hydroxylamine (HA; the positive control for oxime formation), followed by silylation and GC-MS analysis. All three incubations produced the same product (Supplemental Figure S6), which we identified as PLP-oxime based on characteristic MS fragmentations and the molecular ion at *m*/*z* 550.69 (calculated 550.88, Supplemental Figure S7 and Table S5). In addition, we found at least two unidentified cycloserine-specific peaks. PLP-oxime also forms in reactions of enzyme-free PLP with LCS, DCS, or HA (Supplemental Figure S8), again accompanied by several unidentified cycloserine-specific intermediates or products (Supplemental Figure S9).

**Figure 5.**
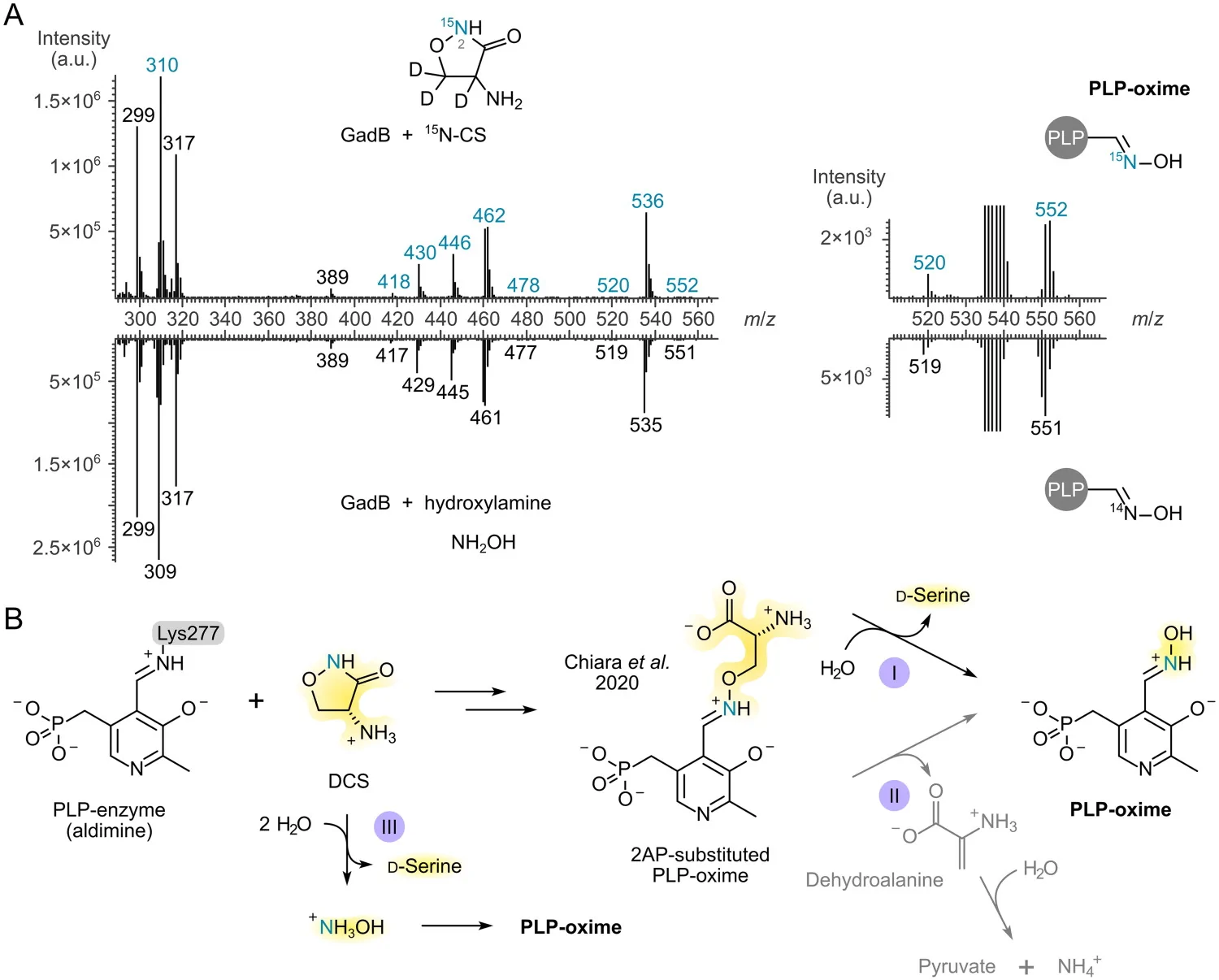
Formation of PLP-oxime from isotopically labelled cycloserine. (A) Mass spectra of the PLP-oxime product obtained after incubating *Mtb*GadB with (top) 2 mM ^15^N-CS or (bottom) 1 mM hydroxylamine as a control; the higher *m*/*z* region is shown separately. Signals corresponding to the incorporation of ^15^N, observed as a + 1 shift in *m*/*z*, are highlighted in blue. A simplified structure of PLP-oxime is shown, indicating the position of ^15^N or ^14^N derived from ^15^N-CS or HA, respectively. (B) Proposed cycloserine-promoted inactivation pathway. The pathway begins with the enzyme-bound PLP-aldimine reacting with d-cycloserine (DCS) before forming PLP-oxime, potentially via a 2-aminopropanoate (2AP)-substituted PLP-oxime intermediate previously described for DCS-treated alanine racemase^23^. The nitrogen atom from DCS traced to PLP-oxime is highlighted in blue.

To determine the origin of the oxime nitrogen, *Mtb*GadB was incubated with isotopically labelled *rac*-[2-^15^N-4,5,5-^2^H_3_]-cycloserine (^15^N-CS) or with HA as a control. Mass spectra show a prominent +1 shift (Figure 5(A)) as well as molecular ions at *m/z* 551.78 (calculated 551.87) for ^15^N-CS-reacted PLP and *m/z* 550.98 for HA-reacted PLP (Supplemental Table S6), demonstrating that the oxime nitrogen originates from the N2 atom of the cycloserine oxazolidine ring.

Based on these data, we propose the following cycloserine-dependent inactivation pathway for *Mtb*GadB. In this model, the amino group of cycloserine displaces Lys277 to form a PLP–cycloserine adduct, which presumably undergoes hydrolysis, other rearrangements, or both, to yield 2-aminopropanoate-substituted PLP-oxime (Figure 5(B)), as previously described for Alr^22^^,^^23^. Unlike in Alr, however, the proposed pathway proceeds further via loss of the 2-aminopropanoate group, producing PLP-oxime with an unsubstituted aldoxime moiety. Theoretically, this process occurs via a hydrolytic route (I), which directly generates serine and PLP-oxime, via a less likely base-assisted elimination route (II) towards pyruvate via the unstable dehydroalanine intermediate (Figure 5(B)), or via another unknown route. Route II was excluded, as no silylated pyruvate was detected in the reaction mixtures (Supplemental Figure S10). Because serine co-elutes with cycloserine (data not shown), we could not unambiguously identify it in the reaction mixtures to provide evidence for route I. Alternatively, DCS could hydrolyse to serine and hydroxylamine (III), which could then directly substitute the Lys277 residue, forming PLP-oxime, similar to control reactions with manually added HA (Supplemental Figure S6). However, no detectable HA was formed in DCS stability assay under varying conditions (pH 4.5–7.5, up to 0.5 mM DCS; data not shown), indicating that route I is likely the major channel towards PLP-oxime.

In summary, our results establish *Mtb*GadB not only as a glutamate decarboxylase that functions optimally under acidic conditions, but also as a hexameric/dimeric PLP-dependent enzyme with strict specificity for l-Glu. We show that *Mtb*GadB is inhibited by both cycloserine enantiomers, with LCS displaying markedly higher inhibitory potency than DCS. Our structural and spectroscopic analysis reveals that cycloserine-mediated inhibition involves loss of the internal PLP–Lys277 aldimine without perturbing the overall hexameric architecture. Instead, inhibition is associated with the formation of PLP-oxime bound within the active site, as confirmed by crystallography, GC-MS, and isotopic labelling experiments that trace the oxime nitrogen to the oxazolidine ring of cycloserine. Together, these findings highlight a previously unrecognised vulnerability of *Mtb*GadB to cycloserine and provide a framework that links PLP chemistry, enzyme oligomerisation, and potentially acid-stress adaptation in *Mycobacterium tuberculosis*.

## Discussion

Survival of *Mtb* within host macrophages requires robust mechanisms to maintain intracellular pH homeostasis in response to phagosomal acidification. Although *Mtb* can partially arrest phagosome maturation^4^, it remains exposed to acidic stress, necessitating active buffering strategies. In this context, glutamate decarboxylase (GadB) has recently emerged as a contributor to mycobacterial acid tolerance^16^. Notably, *Mtb* maintains a near-neutral intracellular pH even when exposed to acidic environments^6^, implying the existence of pH-regulatory mechanisms in which GadB likely plays a role. Our study provides a comprehensive biochemical, structural, and mechanistic characterisation of *Mtb*GadB, highlighting its role not only as a functional acid-active enzyme, but also as a previously unrecognised cycloserine target.

Our biochemical characterisation establishes *Mtb*GadB as an acid-active, strictly l-glutamate (l-Glu)-specific, PLP-dependent GAD. As such, *Mtb*GadB may play a physiological role during intraphagosomal residence. Notably, *Mtb*GadB exhibits moderate substrate inhibition at elevated l-Glu concentrations (Figure 1(A)). The enzyme displays maximal activity between pH values 3.5 and 5.5 (Figure 1(B)), a range consistent with the acidic environment of phagolysosomes (pH ∼4.5) and closely matching the reported optimum of Gad from *Mycobacterium smegmatis* (pH 5.4)^39^ and of other bacterial GADs (Supplemental Table S3). This is in contrast to the pH dependence recently reported for *Mtb*GadB^14^. However, the authors used a narrow range of sodium acetate buffering capacity and a much higher substrate concentration, which may explain this discrepancy.

Given the central role of PLP in GAD catalysis, we hypothesised that cycloserine – a long-standing second-line antitubercular drug known to inhibit the PLP-dependent enzymes alanine racemase and d-alanine:d-alanine ligase^19^^,^^23^ – might also inhibit GadB. To date, only a limited number of GadB inhibitors have been described, primarily for *E. coli* GadB, including aliphatic dicarboxylic acids (pimelate, adipate, and glutarate), l-isoglutamate, and the suicide inhibitor α-(fluoromethyl)glutamate^40–43^. Here, we demonstrate that both cycloserine enantiomers – DCS and LCS – inhibit *Mtb*GadB (Figure 2(A,B)), with LCS approximately ninefold more potent. This correlates with the preference of *Mtb*GadB for l-Glu.

Furthermore, fluorescence spectroscopy provides direct evidence that cycloserine inhibition of *Mtb*GadB interrupts the internal PLP–Lys277 aldimine (Figure 2(C,D)). The time-dependent decrease in fluorescence emission, which closely correlates with enzymatic inhibition, is absent in the Lys277Arg mutant lacking the internal aldimine, confirming that cycloserine acts through PLP chemistry rather than non-specific protein destabilisation. The faster rate observed with LCS further supports its greater inhibitory potency compared to DCS (Figure 2).

High-resolution crystal structures of native and DCS-treated *Mtb*GadB revealed that inhibition is achieved without large-scale conformational rearrangements or disruption of the hexameric assembly. Instead, in accordance with fluorescence spectroscopy data, DCS selectively targets the active site, displacing the Lys277 residue from the PLP-aldimine. In addition, the released coenzyme is converted into a C4’-modified derivative that remains bound in the active site (Figure 4(B)). The absence of electron density, consistent with larger PLP–cycloserine adducts previously described for Alr^23^, indicate a novel inhibition product, likely an oxime, based on the expected two nonhydrogen atoms bound to the C4’ atom of the former PLP.

GC-MS analysis of *Mtb*GadB incubated with the cycloserine enantiomers clearly identified PLP-oxime in the reaction mixtures (Supplemental Figure S7), providing an important foundation for its presence in the active site. Moreover, our isotopic labelling experiments demonstrate that the oxime nitrogen originates from the N2 atom of the cycloserine oxazolidine ring (Figure 5(A)), establishing a direct chemical link between the inhibitor and PLP modification. Interestingly, PLP-oxime also forms in model reactions of enzyme-free PLP with cycloserine enantiomers (Supplemental Figure S8), although the composition of the putative cycloserine-derived intermediates or products slightly differs from that of incubations involving the enzyme (Supplemental Figures S6 and S9). Identification of these compounds was beyond the scope of this work.

Based on the above findings, we propose a cycloserine-mediated inactivation pathway involving several intermediates that may be identical or similar to those described for Alr^23^. These intermediates could also account for the presumably cycloserine-derived compounds detected in the *Mtb*GadB incubations (Supplemental Figure S6). After the initial steps, the proposed pathway could form 2-aminopropanoate (2AP)-substituted PLP-oxime as an intermediate (Supplemental Figure 5B), unlike Alr, where the compound forms the final product^23^. Further reactions might proceed via route I involving hydrolytic 2AP removal (Supplemental Figure 5B), less likely via route II involving 2AP elimination, or by another unknown route. The alternative route III, involving hydroxylamine-forming cycloserine hydrolysis, which circumvents the reaction of cycloserine with PLP, is also less probable. Although DCS, consistent with previous data on its aqueous stability^44^, remains stable under controlled laboratory experiments, we cannot rule out the possible contribution of the intracellular environment to cycloserine hydrolysis via route III, yielding HA and, in turn, inducing GadB inhibition. Future experiments using isotopically labelled solvent and NMR spectroscopy together with analysis of reaction mixtures by LC-MS could help to establish the precise mechanism of GadB inhibition by cycloserine.

Collectively, our findings expand the known target spectrum of cycloserine beyond cell wall-biosynthetic enzymes, identifying GadB as a previously unrecognised PLP-dependent target in *M. tuberculosis*. Although inhibition of GadB alone is unlikely to be bactericidal as the *gadB* gene is non-essential^45^ and the IC_50_ lies in the mid-hundreds of µM range, impairment of GadB-mediated glutamate decarboxylation may compromise intracellular pH homeostasis, particularly in latent acidic niches. The ability of cycloserine to generate distinct PLP adducts in dependence on enzyme microenvironment of the active site highlights the importance of detailed mechanistic studies of next-generation PLP-targeting drugs.

## Supplementary Material

264101224_Supplemental material (1).docx

## Data Availability

Crystallographic data for *Mtb*GadB and its complex with PLP-oxime were deposited in the PDB with codes pdb_00009t6a (holoenzyme; https://doi.org/10.2210/pdb9T6A/pdb) and pdb_00009t6b (DCS-treated enzyme; https://doi.org/10.2210/pdb9T6B/pdb), respectively. Kinetic and chromatographic data are available from the authors upon request.
