# Supplementary material for: Cycloserine inhibits glutamate decarboxylase from Mycobacterium tuberculosis
Source: J Enzyme Inhib Med Chem. 2026 Sep 21;41(1):2707854. doi: 10.1080/14756366.2026.2707854 (PMC13600307; doi:10.1080/14756366.2026.2707854)
Supplement: Supplementary file 1 [file IENZ_A_2707854_SM7274.docx]

**Supplemental material**

Cycloserine inhibits glutamate decarboxylase from *Mycobacterium tuberculosis*

Jan Snášel, Jiří Dostál, Michal Tupec, Ondřej Bulvas, Iva Pichová

Institute of Organic Chemistry and Biochemistry, Czech Academy of Sciences, Prague, Czech Republic

**Figure S1. Sequence conservation among non-eukaryotic aspartate/glutamate decarboxylases, including *Mtb*GadB.** Functionally characterized enzymes^1–9^ and enzymes with experimentally determined structures^9–12^ are indicated. The region corresponding to the structurally conserved β-hairpin is marked by a green line. Alignment numbering refers to the *Mtb*GadB sequence. Species abbreviations, full species names, and accession codes are provided in Table S1.

**Figure S2. *Mtb*GadB purification and its decarboxylase activity.** (A) SDS-PAGE analysis of *Mtb*GadB purification process. Lanes: Protein standard; 1, GadB sample from Ni-NTA resin; 2, GadB sample from Superdex 200 10/300 GL column. (B) Dependence of relative reaction velocity on *Mtb*GadB concentration, illustrating substrate limitation at higher concentrations. Reactions were carried out at pH 4.5 in the presence of 1 mM l-glutamate and 20, 30, 60, or 150 μg/mL GadB, and proceeded for 30 minutes at room temperature.

**Figure S3. Inhibitory effect of copper ions on *Mtb*GadB activity.**

**Figure S4. pH-dependent oligomerization of *Mtb*GadB.** The multimeric state of *Mtb*GadB determined by mass photometry is shown as a function of pH. Each subpanel displays the molecular mass distribution, with fitted Gaussian curves indicating distinct oligomeric species and counts of detected particles representing their relative abundances.

**Figure S5. Dimer interface of native *Mtb*GadB.** (A) Dimeric structure of the enzyme, highlighting the conserved β-hairpin (residues 301–314, red) positioned near the active site (black star) of the adjacent subunit. Cartoon and surface representation were used to distinguish the two subunits. (B) View of the active site located at the interface between the two subunits (colored yellow and blue), including solvent molecules. Conserved residues from Fig. S1 are indicated.

**Figure S6. GC-MS analysis of *Mtb*GadB inactivation products.** Selected regions of total ion chromatograms (TICs) from silylated mixtures of *Mtb*GadB incubated with 2 mM l-cycloserine, 2 mM d-cycloserine, or 1 mM hydroxylamine. Retention times corresponding to PLP-oxime isomers (*E*/*Z*) are indicated by dashed lines, with the respective peaks marked by asterisks. Cycloserine-specific peaks are indicated by circles.

**Figure S7. Mass spectrometric characterization of PLP-oxime.** Mass spectra of a PLP-derived product formed upon incubation of *Mtb*GadB with 2 mM l-cycloserine, 2 mM d-cycloserine, or 1 mM hydroxylamine are shown, together with the structure of silylated PLP-oxime and selected diagnostic fragmentations. The *m*/*z* value corresponding to the molecular ion is highlighted in red.

**Figure S8. GC-MS analysis of PLP reactions.** Selected regions of TICs from silylated mixtures of 20 μM PLP incubated with 2 mM l-cycloserine, 2 mM d-cycloserine, or 1 mM hydroxylamine are shown. Retention times corresponding to PLP-oxime isomers are indicated by dashed lines, with the respective peaks marked by asterisks. Cycloserine-specific peaks are indicated by circles. Peaks labeled 1–3 correspond to those shown in Figure S6.

**Figure S9. Additional intermediates/products.** Mass spectra of the remaining peaks 1–6 from Figures S6 and S8, derived from PLP, cycloserine, or both.

**Figure S10. GC-MS screening for pyruvate.** Selected regions of extracted ion chromatograms (EIC; at *m*/*z* 217+232) from silylated mixtures of *Mtb*GadB incubated with 2 mM l-cycloserine, or 2 mM d-cycloserine, including pyruvate as a positive control. The spectrum of silylated pyruvate is shown together with its diagnostic fragmentations.

**Table S1. List of protein sequences used in Figure S1.**

| **Abbreviation** | **Species** | **Accession code** |
| --- | --- | --- |
| *Mtb* | *Mycobacterium tuberculosis* | WP_003418277 |
| *Lbr* | *Levilactobacillus brevis* | WP_024855475 |
| *Eco* | *Escherichia coli* | AAA23834 |
| *Lla* | *Lactococcus lactis* | BAA24585 |
| *Lzy* | *Levilactobacillus zymae* | AHF72525 |
| *Eav* | *Enterococcus avium* | WP_311892761 |
| *Afi* | *Aliivibrio fischeri* | AAW85559 |
| *Sth* | *Streptococcus thermophilus* | MGZ0941042 |
| *Bth* | *Bacteroides thetaiotaomicron* | AAO77677 |
| Dpb*.* sp*.* | Deltaproteobacteria sp*.* | MBC8177799 |
| *Fan* | *Fusobacterium animalis* | WP_008795652 |
| *Mha* | *Mycoplasma hafezii* | WP_426460892 |
| *Cpo* | *Cloacibacillus porcorum* | WP_066747595 |
| *Tko* | *Thermococcus kodakarensis* | BAD86003 |

**Table S2. List of oligonucleotides used for mutagenesis.** Mutated codons are shown in bold, restriction sites are underlined.

| **Name** | **Sequence (5’ to 3’)** |
| --- | --- |
| 277R_F | TCGATCAACGTCAGCGGCCAC**AGG**TATGGGCTGACCTATCCCGGC |
| 277R_R | GCCGGGATAGGTCAGCCCATA**CCT**GTGGCCGCTGACGTTGATCGA |
| GadB_F | GTGTCTCGCAGTCACCCGTCCGTGCC |
| GadB_R | GTGCGCGAAATGCTGTGCGTCGAAGT |
| NdeI_F | GTACTAGACCATATGGTGTCTCGCAGTCACCCGTCCGTGCC |
| XhoI_R | CTGGACCGTCTCGAGCTAGTGCGCGAAATGCTGTGCGTCGAAGT |

**Table S3. Biochemical properties of selected functionally characterized glutamate decarboxylases from Figure S1, including *Mtb*GadB.**

| **Species** | **Optimal pH** | **Effect of Metal Ions** | | ***K*_M_** | **Monomer Mass** | **Oligomeric State** | **Reference** |
| --- | --- | --- | --- | --- | --- | --- | --- |
|  |  | **Positive** | **Negative** | **mM** | **kDa** |  |  |
| *M. tuberculosis* | 3.5–5.5 | none | Cu^2+^ | 1 | 51 | hexamer | This work |
| *E. coli* | 4.6 | Ca^2+^ | none | 12.8 | 53 | hexamer | 1 |
| *Streptococcus thermophilus* | 4.5 | none | none | 2.7 | 52 | n.d. | 2 |
| *Levilactobacillus zymae* | 4.5 | NH_4_^+^, Ca^2+^, Mg^2+^, Mn^2+^, Na^+^ | Co^2+^, Cu^2+^, Ag^+^ | 1.7 | 53 | n.d. | 3 |
| *Levilactobacillus brevis* | 5.2 | Ca^2+^, Mg^2+^, Mn^2+^, Na^+^ | Ag^+^, Zn^2+^, Cu^2+^, K^+^ | 3.6 | 53 | dimer | 4 |
| *Enterococcus avium* | 4.5 | Ca^2+^, Mg^2+^, Mn^2+^, Zn^2+^ | Cu^2+^, Ag^+^ | 3.3 | 54 | n.d. | 5 |

**Table S4. Data collection and refinement statistics for *Mtb*GadB and its complex with DCS.** Values for the highest-resolution shell are shown in parentheses.

|  | GadB | GadB + DCS |
| --- | --- | --- |
| PDB | pdb_00009t6a | pdb_00009t6b |
| **Crystal data** | | |
| Resolution range (Å) | 36.63–1.41 (1.46–1.41) | 36.28–1.41 (1.44–1.41) |
| Space group | R 3 2 :H | R 3 2 :H |
| Unit cell | 121.834 121.834 193.281 90 90 120 | 121.19 121.19 193.29 90 90 120 |
| **Data collection** | | |
| Wavelength (Å) | 0.97625 | 0.9184 |
| Unique reflections | 105848 (10506) | 104543 (10362) |
| Completeness (%) | 99.98 (99.99) | 99.85 (99.03) |
| Wilson *B*-factor | 13.93 | 19.27 |
| **Refinement statistics** | | |
| Reflections used in refinement | 105848 (10505) | 104543 (10280) |
| Reflections used for R*free* | 1411 (140) | 2094 (136) |
| R*work* | 0.1667 (0.3530) | 0.1655 (0.4642) |
| R_free_ | 0.1822 (0.3759) | 0.1771 (0.4333) |
| Number of non-hydrogen atoms | 3692 | 3821 |
| Macromolecules | 3374 | 3488 |
| Ligands | 42 | 42 |
| Solvents | 276 | 291 |
| Protein residues | 436 | 436 |
| RMS(bonds) | 0.016 | 0.008 |
| RMS(angles) | I.28 | 0.97 |
| **Ramachandran plot** | | |
| Ramachandran favored (%) | 97.70 | 97.47 |
| Ramachandran allowed (%) | 2.30 | 2.53 |
| Ramachandran outliers (%) | 0.00 | 0.00 |
| Rotamer outliers (%) | 0.52 | 0.80 |
| Clashscore | 1.48 | 2.71 |
| Average *B*-factor | 25.27 | 26.15 |

**Table S5. MS data used to calculate the weighted average *m*/*z* of the silylated PLP-oxime molecular ion.** Spectra originate from samples of *Mtb*GadB incubated with l-cycloserine, d-cycloserine, or hydroxylamine (*n* = 3).

| **GadB + l-cycloserine** | | | | | |
| --- | --- | --- | --- | --- | --- |
| **Sample 1** | | **Sample 2** | | **Sample 3** | |
| ***m*/*z*** | **Intensity (a.u.)** | ***m*/*z*** | **Intensity (a.u.)** | ***m*/*z*** | **Intensity (a.u.)** |
| 549.039849 | 1888.328990 | 549.041462 | 1622.043762 | 549.227538 | 1500.866883 |
| 550.099041 | 5869.330635 | 550.065723 | 5063.154675 | 550.170155 | 4151.962727 |
| 551.122082 | 4502.286365 | 551.084287 | 3660.416412 | 551.121718 | 2410.529473 |
| 552.109045 | 1820.990505 | 552.124410 | 1376.691332 | 552.130841 | 977.549931 |
| 553.128432 | 314.638902 | 553.050387 | 379.019810 | 553.058787 | 478.662734 |
| 554.247435 | 180.124390 |  |  | 555.094149 | 23.526852 |
| 555.169523 | 78.843916 |  |  |  |  |
| **Weighted average *m*/*z*** | | | | | |
| 550.669942 | | 550.564213 | | 550.620137 | |

| **GadB + d-cycloserine** | | | | | |
| --- | --- | --- | --- | --- | --- |
| **Sample 1** | | **Sample 2** | | **Sample 3** | |
| ***m*/*z*** | **Intensity (a.u.)** | ***m*/*z*** | **Intensity (a.u.)** | ***m*/*z*** | **Intensity (a.u.)** |
| 549.106748 | 2970.143016 | 549.074628 | 3857.768906 | 549.096649 | 4196.986914 |
| 550.071709 | 8966.168466 | 550.080027 | 9290.965094 | 550.096946 | 10291.228790 |
| 551.065351 | 9062.869227 | 551.120761 | 9144.446947 | 551.079530 | 8335.146940 |
| 552.069446 | 2859.767631 | 552.095662 | 3961.391275 | 552.097071 | 4373.702985 |
| 553.067504 | 1023.288915 | 553.080874 | 1069.246460 | 553.047834 | 1491.487600 |
| 554.115248 | 210.909003 | 554.142693 | 548.263780 | 554.137018 | 564.672039 |
| 555.153452 | 69.639189 |  |  | 555.097620 | 446.195994 |
| **Weighted average *m*/*z*** | | | | | |
| 550.712517 | | 550.763834 | | 550.826035 | |

| **GadB + hydroxylamine** | | | | | |
| --- | --- | --- | --- | --- | --- |
| **Sample 1** | | **Sample 2** | | **Sample 3** | |
| ***m*/*z*** | **Intensity (a.u.)** | ***m*/*z*** | **Intensity (a.u.)** | ***m*/*z*** | **Intensity (a.u.)** |
| 549.056153 | 4839.664948 | 549.091402 | 4834.531385 | 549.116407 | 4711.538336 |
| 550.078907 | 12259.698290 | 550.046881 | 14995.271346 | 550.065187 | 13639.235065 |
| 551.084337 | 9193.372009 | 551.048041 | 12312.781705 | 551.078390 | 11857.033653 |
| 552.073366 | 3730.586509 | 552.010989 | 4336.958728 | 552.072975 | 3984.621759 |
| 553.108892 | 1291.021395 | 553.033300 | 1265.307253 | 553.088787 | 1649.132446 |
| 554.089500 | 523.296874 | 553.985873 | 331.116095 | 554.116399 | 420.916083 |
| 555.151851 | 509.978141 | 555.389812 | 158.193082 | 555.321573 | 197.007774 |
|  |  |  |  | 555.928255 | 283.192135 |
| **Weighted average *m*/*z*** | | | | | |
| 550.707442 | | 550.626317 | | 550.743720 | |

**Table S6. MS data used to calculate the weighted average *m*/*z* of the silylated PLP-oxime molecular ion.** Spectra originate from samples of *Mtb*GadB incubated with *rac*-^15^N,d_3_-cycloserine (*n* = 3) or hydroxylamine (*n* = 2).

| **GadB + *rac*-^15^ N,d_3_ -cycloserine** | | | | | |  | **GadB + hydroxylamine** | | | |
| --- | --- | --- | --- | --- | --- | --- | --- | --- | --- | --- |
| **Sample 1** | | **Sample 2** | | **Sample 3** | |  | **Sample 1** | | **Sample 2** | |
| ***m*/*z*** | **Intensity (a.u.)** | ***m*/*z*** | **Intensity (a.u.)** | ***m*/*z*** | **Intensity (a.u.)** |  | ***m*/*z*** | **Intensity (a.u.)** | ***m*/*z*** | **Intensity (a.u.)** |
| 549.042035 | 176.928144 | 549.572998 | 0.000000 | 550.041486 | 1944.238473 |  | 549.117612 | 5289.941452 | 549.145221 | 4642.634390 |
| 550.175721 | 2617.697640 | 550.357389 | 976.163610 | 550.564209 | 90.251732 |  | 550.124710 | 14211.351604 | 550.104077 | 13103.388664 |
| 551.161969 | 4985.827825 | 551.152411 | 4638.655273 | 551.102047 | 6709.358088 |  | 551.123447 | 18913.330841 | 551.126898 | 19030.837831 |
| 552.128632 | 4434.779255 | 552.128313 | 5605.565012 | 552.119863 | 7519.452124 |  | 552.099187 | 8370.998075 | 552.108947 | 8226.208198 |
| 553.080093 | 2357.958113 | 553.141758 | 2654.412937 | 553.138732 | 1933.532856 |  | 553.105203 | 3014.398115 | 553.165139 | 3145.383975 |
| 554.131962 | 295.208081 | 554.133438 | 469.299037 | 554.187518 | 127.177908 |  | 553.972779 | 550.752065 | 554.044870 | 801.888905 |
| 555.397453 | 7.093078 | 555.325821 | 170.236629 | 555.217397 | 527.333076 |  | 555.122817 | 30.656815 | 554.620004 | 72.312228 |
|  |  |  |  |  |  |  |  |  | 555.242545 | 105.245583 |
| **Weighted average *m*/*z*** | | | | | |  | **Weighted average *m*/*z*** | | | |
| 551.616396 | | 551.984996 | | 551.740912 | |  | 550.945394 | | 551.023344 | |

**Supplemental references**

1. Thu Ho NA, Hou CY, Kim WH, Kang TJ. Expanding the active pH range of *Escherichia coli* glutamate decarboxylase by breaking the cooperativness. J Biosci Bioeng. 2013;115:154–158. DOI: https://doi.org/10.1016/j.jbiosc.2012.09.002.
2. Zhang C, Lu J, Chen L, Lu F, Lu Z. Biosynthesis of γ-aminobutyric acid by a recombinant *Bacillus subtilis* strain expressing the glutamate decarboxylase gene derived from *Streptococcus salivarius* ssp. *thermophilus* Y2. Proc Biochem. 2014;49:1851–1857. DOI: https://doi.org/10.1016/j.procbio.2014.08.007.
3. Park JY, Jeong SJ, Kim JH. Characterization of a glutamate decarboxylase (GAD) gene from *Lactobacillus zymae*. Biotechnol Lett. 2014;36:1791–1799. DOI: https://doi.org/10.1007/s10529-014-1539-9.
4. Seo MJ, Nam YD, Lee SY, Park SL, Yi SH, Lim SI. Expression and characterization of a glutamate decarboxylase from *Lactobacillus brevis* 877G producing gamma-aminobutyric acid. Biosci Biotechnol Biochem. 2013;77:853–856. DOI: https://doi.org/10.1271/bbb.120785.
5. Lee KW, Shim JM, Yao Z, Kim JA, Kim HJ, Kim JH. Characterization of a glutamate decarboxylase (GAD) from *Enterococcus avium* M5 isolated from Jeotgal, a Korean fermented seafood. J Microbiol Biotechnol. 2017;27:1216–1222. DOI: https://doi.org/10.4014/jmb.1701.01058.
6. Nomura M, Nakajima I, Fujita Y, Kobayashi M, Kimoto H, Suzuki I, Aso H. *Lactococcus lactis* contains only one glutamate decarboxylase gene. Microbiology. 1999;145:1375–1380. DOI: https://doi.org/10.1099/13500872-145-6-1375.
7. Pan S, Nikolakakis K, Adamczyk PA, Pan M, Ruby EG, Reed JL. Model-enabled gene search (MEGS) allows fast and direct discovery of enzymatic and transport gene functions in the marine bacterium *Vibrio fischeri*. J Biol Chem. 2017;292(24):10250–10261. DOI: https://doi.org/10.1074/jbc.M116.763193.
8. Tomita H, Yokooji Y, Ishibashi T, Imanaka T, Atomi H. An archaeal glutamate decarboxylase homolog functions as an aspartate decarboxylase and is involved in β-alanine and coenzyme A biosynthesis. J Bacteriol. 2014;196(6):1222–1230. DOI: https://doi.org/10.1128/jb.01327-13.
9. Liu S, Wen B, Du G, Wang Y, Ma X, Yu H, Zhang J, Fan S, Zhou H, Xin F. Coordinated regulation of *Bacteroides thetaiotaomicron* glutamate decarboxylase activity by multiple elements under different pH. Food Chem. 2023;403:134436–134436 DOI: https://doi.org/10.1016/j.foodchem.2022.134436.
10. Huang J, Fang H, Gai ZC, Mei JQ, Li JN, Hu S, Lv CJ, Zhao WR, Mei LH. *Lactobacillus brevis* CGMCC 1306 glutamate decarboxylase: Crystal structure and functional analysis. Biochem Biophys Res Commun. 2018;503:1703–1709. DOI: https://doi.org/10.1016/j.bbrc.2018.07.102.
11. Capitani G, De Biase D, Aurizi C, Gut H, Bossa F, Grutter MG. Crystal structure and functional analysis of *Escherichia coli* glutamate decarboxylase. EMBO J. 2003;22:4027–4037. DOI: https://doi.org/10.1093/emboj/cdg403.
12. Su CC, Lyu M, Morgan CE, Bolla JR, Robinson CV, Yu EW. A ‘Build and Retrieve’ methodology to simultaneously solve cryo-EM structures of membrane proteins. Nat. Methods. 2021;18:69–75. DOI: https://doi.org/10.1038/s41592-020-01021-2.
